# Exploring the Ovine Anatomy: A Comprehensive Study of the Sheep’s Head for Basic Training in Functional Endoscopic Sinus Surgery

**DOI:** 10.7759/cureus.53529

**Published:** 2024-02-03

**Authors:** Constantin Stan, Cristina Blebea, Mihai I Tănase, Peter L Ujvary, Mara Tănase, Septimiu Sever Pop, Alma A Maniu, Marcel Cosgarea, Doinel G Rădeanu

**Affiliations:** 1 Department of ENT, “Iuliu Haţieganu” University of Medicine and Pharmacy, Cluj-Napoca, ROU; 2 Department of Surgical Clinical, “Lower Danube” University, Faculty of Medicine and Pharmacy, Galati, ROU

**Keywords:** training, surgery, endoscopy, ovine model, simulation

## Abstract

Introduction: Training young doctors in functional endoscopic sinus surgery requires dedicated centers for cadaveric dissections. However, ethical constraints have limited cadaver availability. Alternative anatomical models, like the ovine model, are being explored for effective training, offering easier procurement and resembling human head anatomy. This study aims to demonstrate that the ovine model is useful for endoscopic sinus surgery training, highlighting the anatomical, imaging, histological, and endoscopic aspects.

Methods: Three adult Native Romanian Turcana sheep's heads were obtained fresh and frozen from a local slaughterhouse. Using a helical scanner, CT scans were performed, and anatomical structures in the images were carefully labeled. Two heads frozen at -20°C were serially sectioned, with one cut sagittally, dividing the skull, and the other head sectioned transversely with 2.5 cm thickness. Sectional photographs were taken. The third sheep's head underwent endoscopy, and samples from the septal mucosa and inferior turbinate were collected for histopathology examination. The specimens were processed, stained, and examined by a pathologist.

Results: The study successfully highlighted the gross anatomy, CT imaging aspects, histological characteristics of sheep nasal mucosa, and endoscopic features, demonstrating the similarity of the sheep's head to human anatomy, making it a suitable anatomical training model for endoscopic sinus surgery.

Conclusion: The use of sheep's heads as substitutes for human cadaver heads in nasal surgery simulations presents a promising avenue for research. The anatomical similarities and cost-effectiveness make sheep's heads a practical choice for certain aspects of nasal surgery investigation. However, researchers must approach this methodology with a thorough understanding of its limitations, including anatomical and biomechanical differences. Validation studies comparing outcomes with human models are crucial to establishing reliability. The sheep’s head anatomical model provides a highly valuable experience for young trainees in endoscopic sinus surgery. Despite encountering several challenges, including some anatomical differences, considering its advantageous attributes renders it an ideal material for mimicking surgical procedures in functional endoscopic sinus surgery.

## Introduction

Functional endoscopic sinus surgery (FESS) involves removing tissue that obstructs the ostiomeatal complex and enhancing drainage, all while preserving the regular, unobstructed anatomy and mucous membrane. The rigid fiberoptic nasal telescope ensures excellent visualization of the ostiomeatal complex during surgery, allowing precise focus on critical areas. The captured image can be displayed on a monitor using a small camera attached to the endoscope's eyepiece. Endoscopic sinus surgery is a commonly employed, secure, and efficient remedy for paranasal sinus disorders. The use of powered instrumentation and stereotactic image-guided surgery has enhanced the effectiveness and safety of this procedure. Endoscopic methods for addressing benign tumors in the nose, sinuses, anterior cranial fossa, and orbit are gaining widespread acceptance. Nonetheless, the consideration of potential complications raises the need for comprehensive surgical training in this specialized field [1].

The training of young doctors in the field of FESS requires dedicated training centers where endoscopic sinus dissections can be performed on cadaver heads. Unfortunately, over time, due to ethical considerations, the procurement of cadavers for surgical training has become limited, sometimes even prohibited. In this sense, there is a need for other anatomical models, easier to procure and use, which can be useful for training purposes in the field of FESS.

The ovine model appears in the discussion and is increasingly used for training and simulation in ENT surgery and other surgical fields [2,3] with good results. In endoscopic sinus surgery, there are several authors, including our team [4-9], who proposed the ovine model for surgical training and with very good results in its use.

Endoscopic ear surgery has effectively incorporated the ovine model for the training of aspiring young doctors. The comparison of sheep ear anatomy to that of humans served as a basis for conducting various otological surgical procedures like canaloplasty, myringoplasty, and ossiculoplasty [10]. Another study utilized the ovine model to assess the learning curve of laser stapedotomy in trainees, revealing notable improvements in operative time with repeated practice [11].

In laryngology, the ovine model served as a valuable resource for training in open laryngotracheal surgery. The head and neck were utilized to replicate a range of surgical procedures, including tracheotomy, laryngoplasty, tracheal resection with tracheal sutures, and laryngectomy with pharyngeal sutures. This application of the ovine model proved successful in training for open surgical techniques, offering the distinct advantage of acquiring surgical skills and refining tissue handling on a model closely resembling human anatomy [12].

The successful utilization of the ovine model extended to training in sialendoscopy. A pioneering training program in salivary gland endoscopy was established, employing the sheep's head [13]. Through this study, we aim to highlight gross anatomy, CT imaging aspects, histological characteristics of the sheep's nasal mucosa, and endoscopic features, highlighting the pros and cons of the model to be used as an anatomical training model in endoscopic sinus surgery.

## Materials and methods

In this investigatory study, three mature Native Romanian Turcana sheep, aged above 10 months, were sourced from a local slaughterhouse. The specimens, comprising the heads of the sheep, were acquired in both fresh and frozen states. Examination of these specimens was conducted employing a helical scanner (Siemens Somatom Scope KVP 130kV, slice thickness 1.5 mm), and subsequent CT scans were stored in DICOM format for analysis using the Radiant DICOM Viewer 2022.1.1. Selected CT images, corresponding to anatomical cross-sections, underwent meticulous labeling of all identified anatomical structures.

After CT imaging, two frozen heads preserved at -20°C underwent serial sectioning using an electric saw. One head was sagittally sectioned by meticulously removing the skin with a scalpel and dissection forceps, followed by a sagittal cut, dividing the skull into two equal parts. This procedure maintained the nasal septum on one side while exposing the side wall of the nasal fossa on the other. Additionally, transverse sections with approximately 2.5 cm thickness were obtained from the second frozen head, and corresponding sectional photographs were meticulously documented.

The third sheep's head was evaluated through endoscopic examination and maneuvers, using Karl Storz TelePack X and Karl Storz rigid Hopkins telescopes 0° and 30°. Instruments used for endoscopic maneuvers included Blakesley nasal forceps, backbiter antrum punch, Cottle dorsal scissors, Freer elevator, sickle knife, curved curette, Frazier suction tube, and scalpel handle with no. 11 blade.

During this procedure, samples from the septal mucosa and inferior turbinate were meticulously collected, preserved in formalin, and subsequently transferred to the pathology department for histologic examination. Hematoxylin and eosin staining were employed for the histological sections, and examination was conducted using a Leica DM2500 LED microscope. Photomicrographs were captured using a Leica DMC 2900 digital system. To ensure accuracy and precision, histological assessments were performed by an experienced pathologist, minimizing the potential for errors.

The ethics committee of the “Iuliu Haţieganu” University of Medicine and Pharmacy approved the study (AV258/28.02.2022). Rigorous quality control measures were implemented through the utilization of fresh samples, mitigating the likelihood of errors or biases in the conducted research.

## Results

Gross anatomy

**Figure 1:**
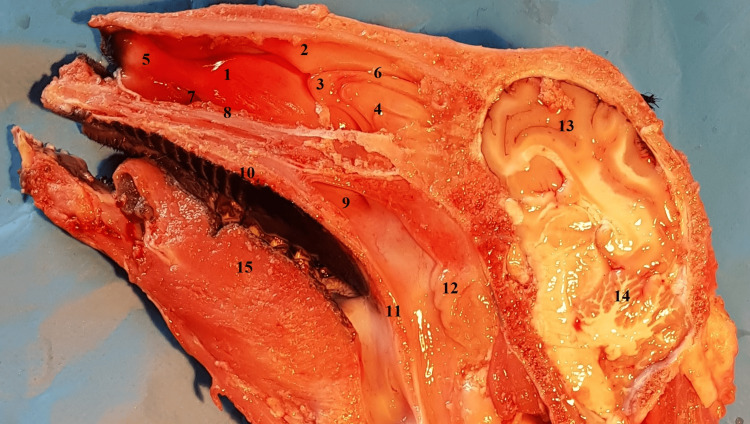
Sagittal midline anatomical section view of the right lateral nasal wall 1: inferior turbinate; 2: superior turbinate; 3: middle turbinate head; 4: ethmoidal labyrinth; 5: anterior part of inferior turbinate; 6: middle nasal meatus; 7: inferior nasal meatus; 8: the palatine process of the maxilla and horizontal plate of the palatine bone; 9: choana; 10: hard palate; 11: soft palate; 12: adenoids; 13: brain; 14: cerebellum; 15: lingual muscles.

Examination of a sagittal anatomical section carried out along the midline of the sheep's head (Figure 1) revealed a series of anatomical structures on the lateral nasal wall.

In the upper portion of the nasal fossa, an anatomical structure can be observed with an anterior portion, which becomes pneumatized posteriorly and may be considered the equivalent of the superior nasal turbinate in humans. It is positioned close to the ethmoidal labyrinth, located superior and anterior to it, and inferomedial to the frontal sinus.

On the same sagittal section, another anatomical structure can be observed that closely resembles the inferior turbinate in humans. Noteworthy, a characteristic aspect of the lower turbinate in sheep is its bistructural component, one upper part, and the other lower part. The middle nasal turbinate, located much deeper, can be partially visualized behind the inferior turbinate. The middle nasal turbinate, as in humans, is an important landmark for identifying the natural ostium of the maxillary sinus.

**Figure 2:**
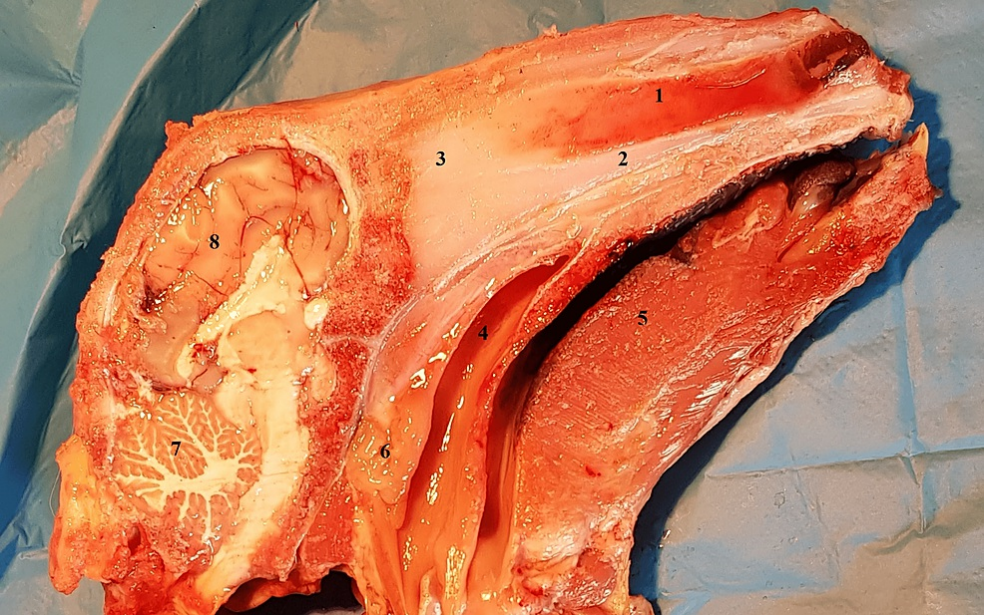
Sagittal midline anatomical section view of the septal nasal wall 1: septal cartilage; 2: vomer bone; 3: perpendicular plate of ethmoid; 4: septal defect; 5: lingual muscles; 6: adenoids; 7: cerebellum; 8: brain.

At the level of the opposite sagittal anatomical section (Figure 2), the nasal septum can be observed. It has a similar appearance to the human nasal septum, with one exception: in its posterior portion, there is an interrupted portion, similar to a human septal defect. More posteriorly, at the level of the nasopharynx, the adenoids that mask the opening of the Eustachian tube can be observed.

**Figure 3:**
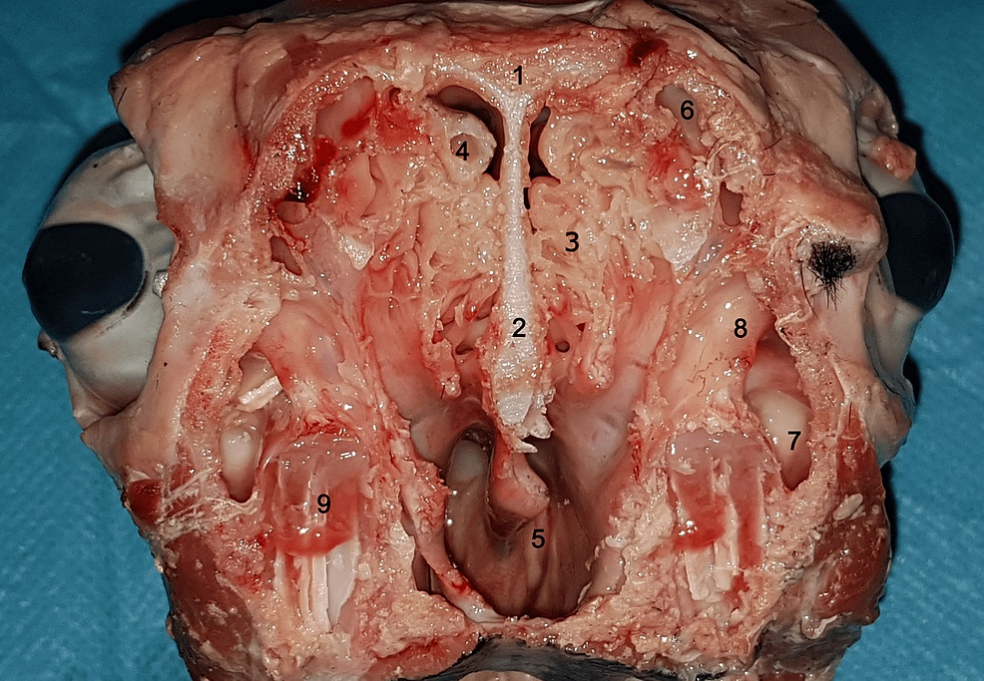
Coronal anatomic section 1: frontal bone; 2: nasal septum; 3: ethmoidal labyrinth; 4: pneumatized posterior portion of the superior turbinate; 5: choana; 6: frontal sinus; 7: maxillary sinus; 8: infraorbital canal; 9: third molar tooth.

Within the context of the ovine model, it is worth highlighting the pneumatization of the ethmoid sinus, which presents good features for practical exercises (Figure 3). This distinctive characteristic provides an excellent opportunity to replicate and simulate the excision of numerous ethmoid cells. The ovine ethmoid sinus, with its well-developed pneumatization, offers a great platform for surgical skills training, as it closely resembles the ethmoid sinus observed in humans. Consequently, this specific feature of the ovine model offers practitioners a valuable training ground to practice ethmoid endoscopic procedures.

Imaging study

**Figure 4:**
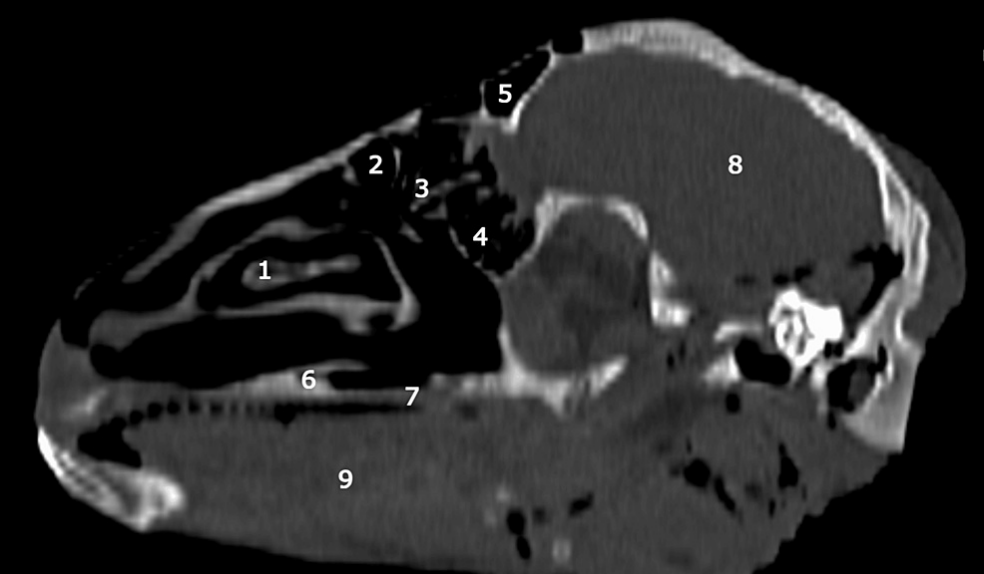
Sagittal CT scan: lateral nasal wall with a high degree of pneumatization of the nasal turbinates and ethmoid sinus 1: inferior turbinate; 2: superior turbinate;3: head of middle turbinate; 4: ethmoidal labyrinth;5: frontal sinus 6: the palatine process of incisive bone; 7: hard palate; 8: brain; 9: body of the tongue.

**Figure 5:**
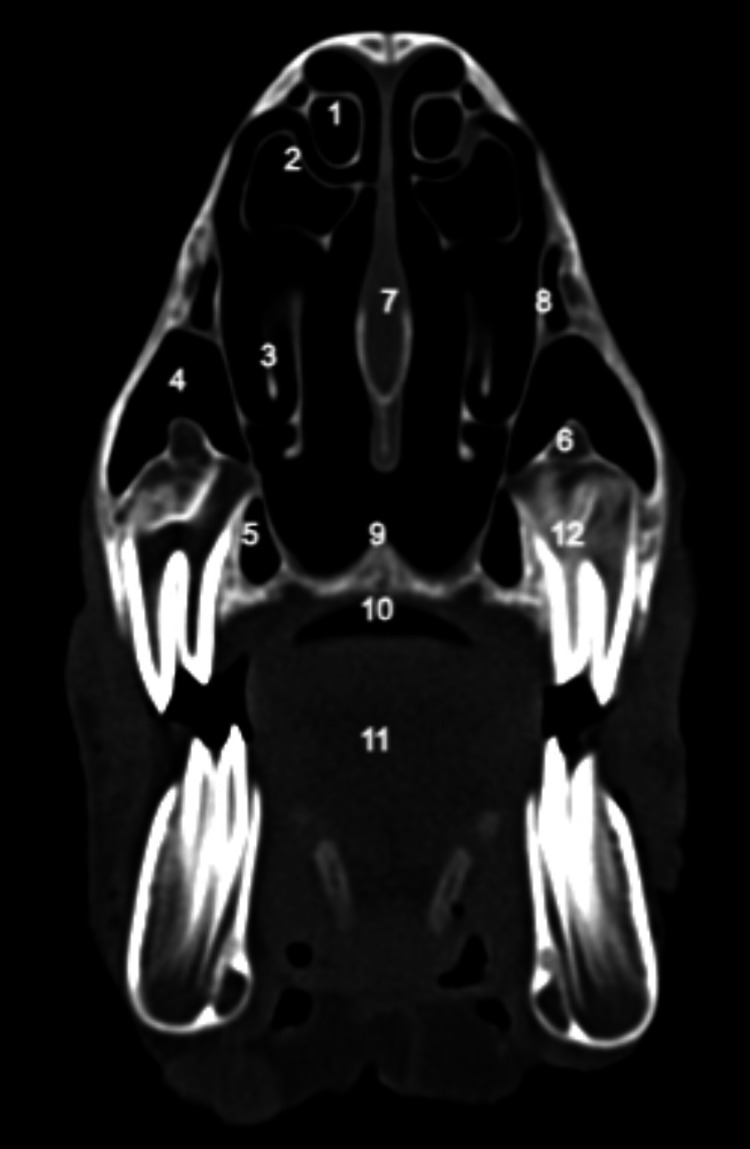
Coronal CT scan at the level of the second molar tooth 1: pneumatized superior turbinate; 2: pneumatized middle turbinate; 3: inferior turbinate with its superior component; 4: lateral chamber of maxillar sinus; 5: medial chamber of maxillar sinus; 6: infra: orbital canal; 7: nasal septum; 8: superior chamber of maxillar sinus; 9: the palatine process of maxilla; 10: hard palate; 11: body of the tongue; 12: second molar tooth.

**Figure 6:**
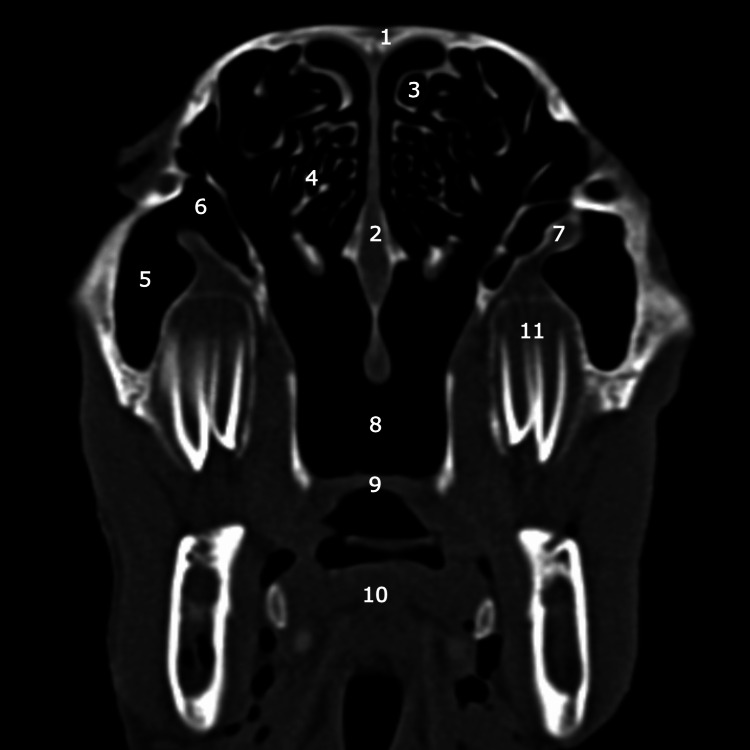
Coronal CT scan at the level of the third molar tooth 1: frontal bone; 2: nasal septum;3: pneumatized superior turbinate; 4: ethmoidal labyrinth;5: lateral chamber of maxillar sinus; 6: medial chamber of maxillar sinus; 7: infra: orbital canal; 8: posterior septal defect; 9: hard palate; 10:  body of tongue; 11: third molar tooth.

On CT imaging, certain aspects can be observed that differ from what is known of human anatomy. The maxillary sinus is not made up of a single chamber, but it has a characteristic three-chamber appearance, due to the existence of three distinct sinuses in its component (Figures 4-6).

Notably, the superior chamber occupies a position above the other two, with a discernible separation facilitated by a bony blade containing the infraorbital nerve, which encounters an interruption in its anterior portion. The natural drainage ostium of the maxillary sinus is located at the level of the superior chamber, while the remaining two chambers ventilate and drain through this superior chamber.

The ethmoidal labyrinth is situated inferior to the superior turbinate, exhibiting pronounced pneumatization in comparison to conventional human anatomical features, characterized by a multitude of air cells.

**Figure 7:**
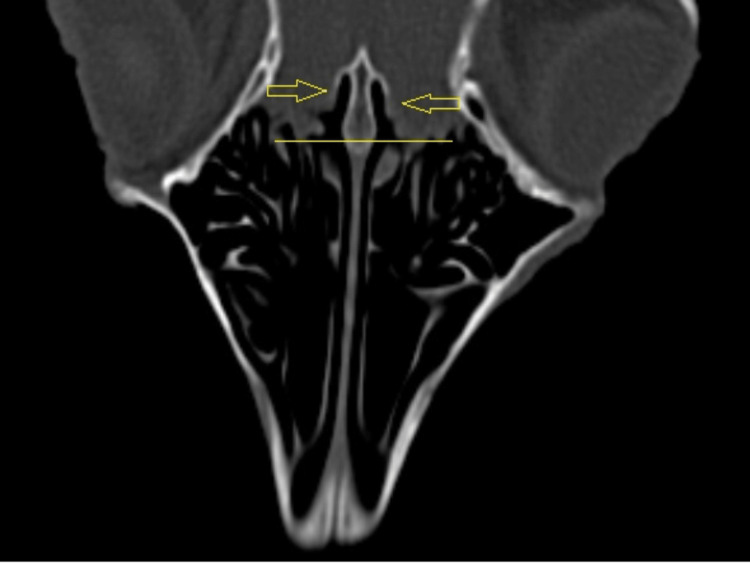
Coronal CT scan: level of the ethmoidal labyrinth The ethmoidal roof corresponds to Keros type 3 from human anatomy.

**Figure 8:**
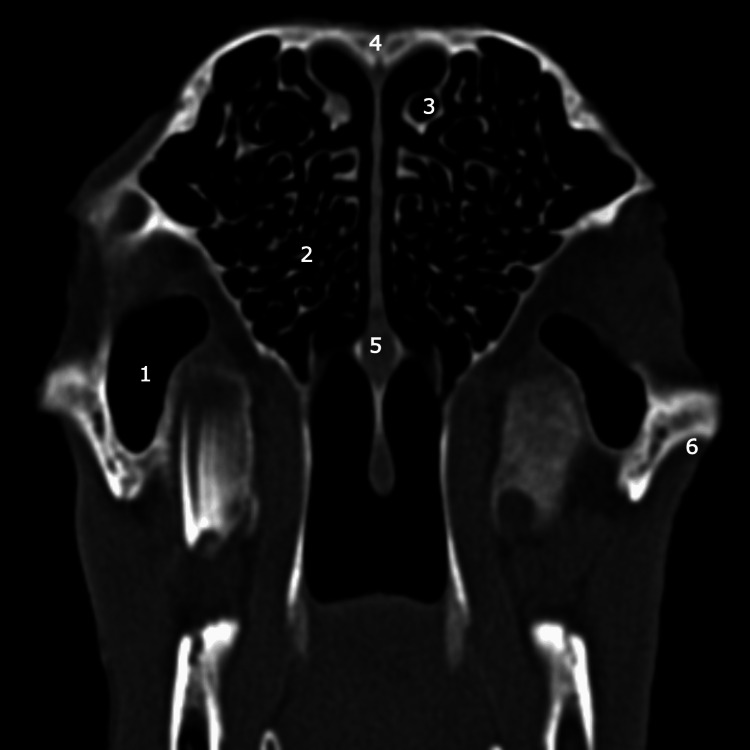
Coronal CT scan: level of the ethmoidal labyrinth 1: maxillar sinus; 2: ethmoidal labyrinth; 3: posterior part of superior turbinate; 4: frontal bone; 5: nasal septum; 6: zigomatich bone.

On coronal CT sections (Figures 7, 8), the characteristic appearance of the ethmoidal roof can be observed, classified as type 3 according to the Keros classification, having a depth of the olfactory fossa of 10 mm.

Another particularity identified both with the help of CT imaging and by dissection, worthy of consideration, is the absence of the sphenoid sinus, an important limitation of the ovine model that must be taken into account at the time of endoscopic sinus surgery training.

**Figure 9:**
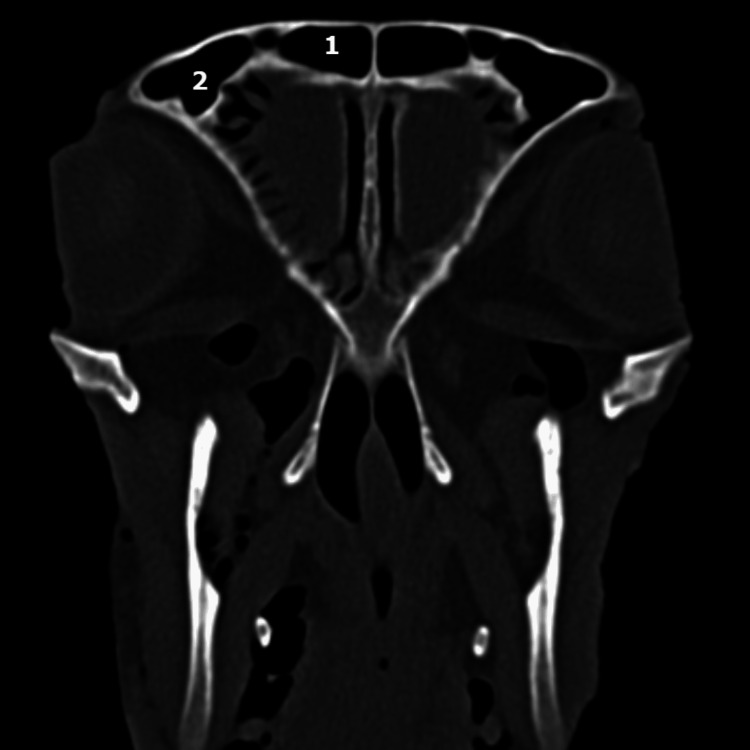
Coronal CT scan: frontal sinus 1: medial chambers of frontal sinus; 2: lateral chambers of frontal sinus.

The frontal sinus also presents a particular aspect specific to the ovine model (Figure 9). It is located above the orbit in general and is formed by a group of chambers arranged circularly from anterior to posterior, determining an aspect that somewhat resembles a crown. All these chambers of the frontal sinus are interconnected for proper ventilation and drainage.

Histology

Samples of both the septal and inferior nasal turbinate mucosa were collected and sent for examination. Both types of mucosa exhibited similar organizational patterns. They featured a surface layer of respiratory epithelium, followed by a loose stroma containing sero-mucous glands. Notably, when compared to human tissue, the sero-mucous glands in the sheep mucosa appeared to be more prominently represented, exhibiting larger and more frequent lobules. The vascularization within the sheep's nasal mucosa is more pronounced compared to other species, including humans. This was particularly notable in the region of the inferior turbinate, where a more polypoid mucosal lining was also observed.

**Figure 10:**
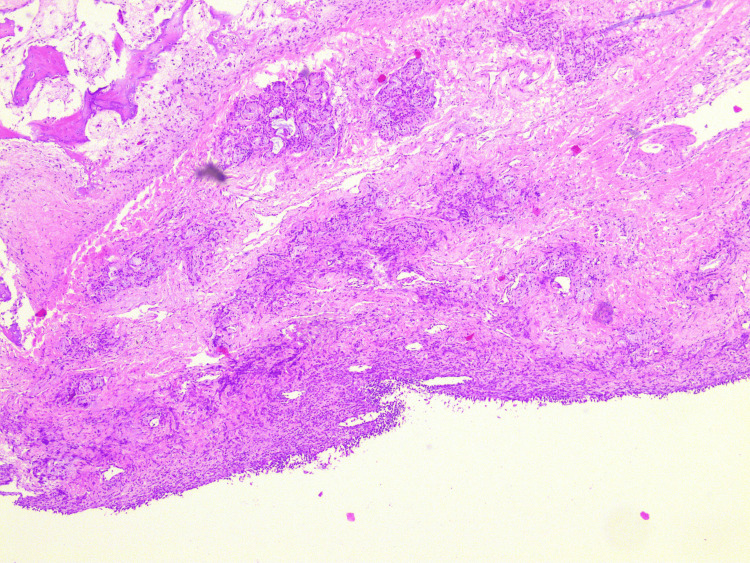
Histological appearance of the ovine septal mucosa HE x50

**Figure 11:**
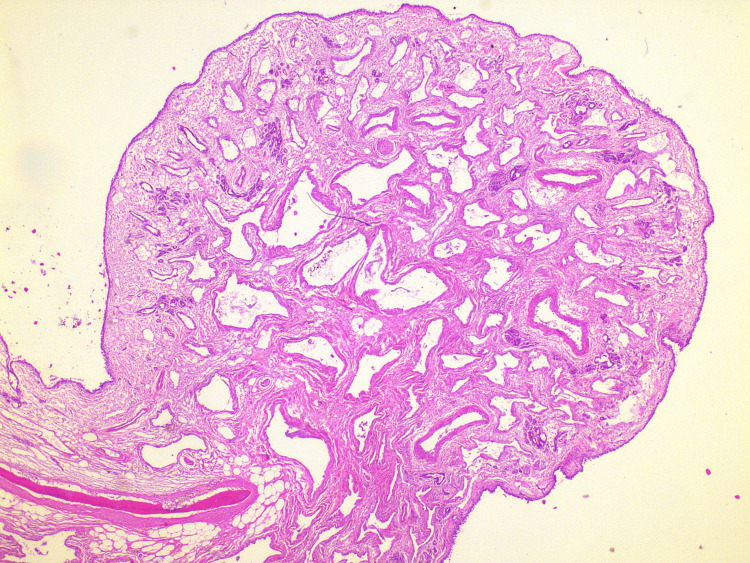
Histological appearance of the ovine inferior nasal turbinate HE x25

Apart from the commonly encountered features of inflammation, congestion, and edema often observed in specific human pathologies, a notable similarity exists in the overall architecture and composition of the mucosa when compared to our understanding of human nasal tissue. The histological examination of the sheep samples revealed a striking resemblance in terms of mucosal structure and its constituent components (Figures 10, 11).

Endoscopic anatomy

**Figure 12:**
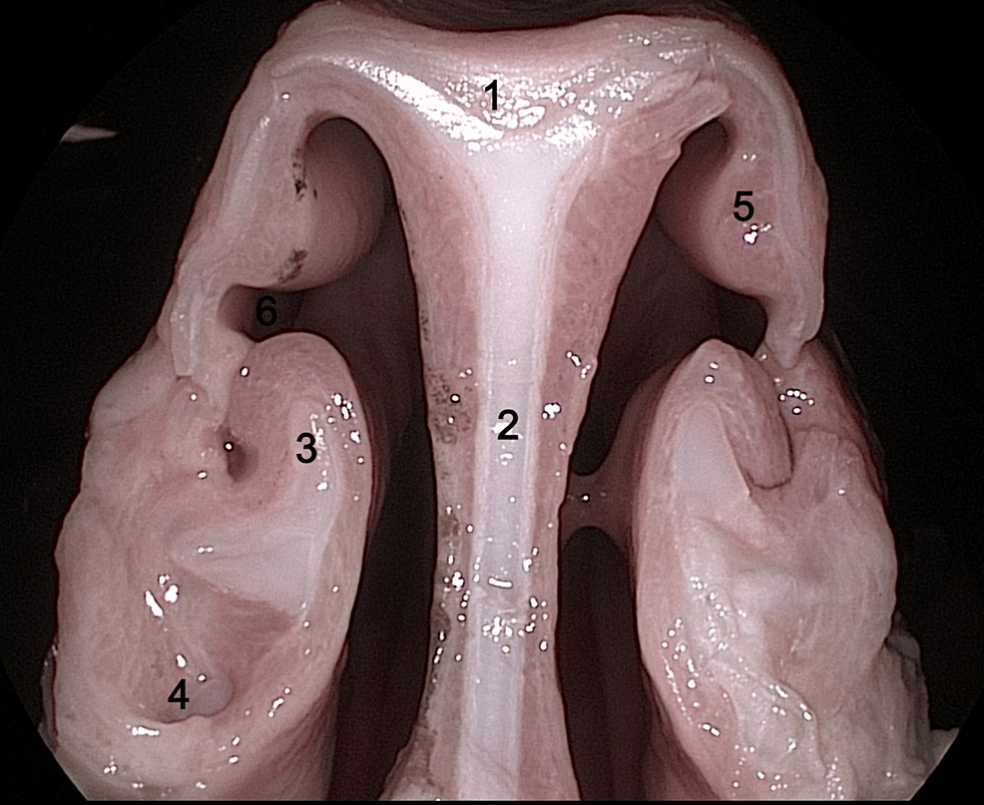
Anterior view of the nasal cavities 1: nasal bone; 2: septal cartilage; 3: inferior turbinate with its superior component; 4: inferior turbinate with its inferior component; 5: anterior portion of the superior turbinate; 6: middle nasal meatus.

In order to optimize the endoscopic approach, a deliberate adjustment was made by slightly shortening the muzzle, a maneuver that served as a practical means to optimize the endoscopic approach, ensuring optimal outcomes and reducing potential challenges associated with restricted access or limited field of view (Figure 12).

**Figure 13:**
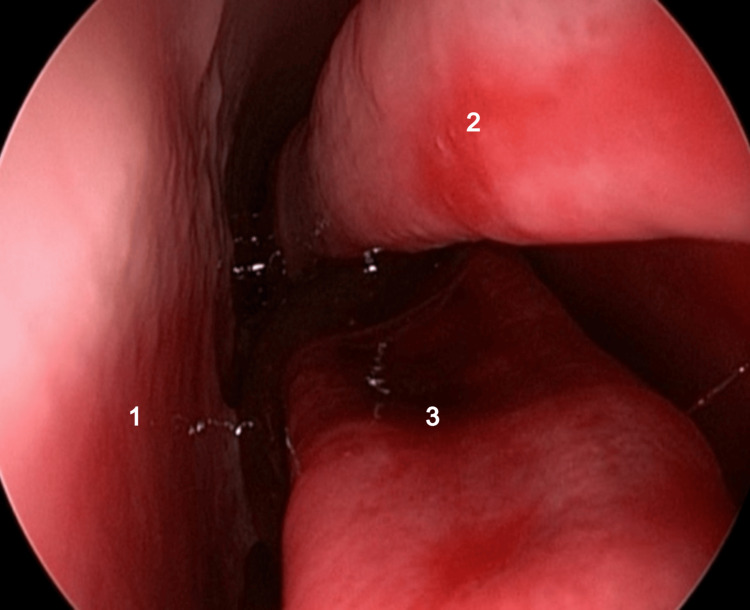
Endoscopic view of the sheep's left nasal fossa 1: septum; 2: superior part of the inferior turbinate; 3: inferior part of the inferior turbinate.

Once the endoscope is inserted into one of the nasal cavities, a resemblance to the anatomical elements of the human nasal cavity becomes apparent. Notably, a sequence of structures can be observed, starting with the nasal septum, which closely mirrors its counterpart in humans in terms of positioning and configuration. Following the nasal septum, there is a distinct structure that bears a remarkable similarity to the inferior turbinate found in human nasal cavities. This structure exhibits comparable features and spatial relationships, contributing to the overall resemblance between the ovine and human nasal anatomies (Figure 13).

As observed on the anatomical sagittal section, a notable anatomical peculiarity can also be observed endoscopically in the inferior turbinate, as it exhibits a distinctive configuration characterized by two primary portions. These two distinct sections are further categorized as the upper portion and the lower portion.

**Figure 14:**
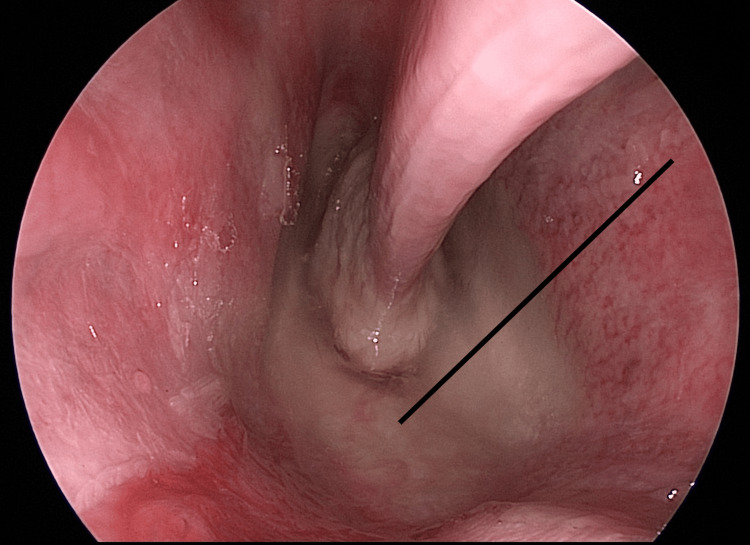
Endoscopic view of the posterior septal defect (continuous black line)

As we progress further with the endoscope within the nasal fossa, an additional distinct characteristic comes into view, namely a posterior septal defect. This specific anatomical feature presents itself as an observable deviation from the norm (Figure 14).

The identification of a posterior septal defect during endoscopic exploration of the nasal fossa highlights an intriguing variation in nasal anatomy. Unlike the typical intact structure of the nasal septum, this particular defect manifests as an absence or discontinuity in the posterior region.

Within the context of the ovine model, the middle turbinate represents a distinct structure situated in the depths of the nasal cavity, requiring specific measures to be observed to visualize it. Notably, its observation becomes possible only when an inferior turbinectomy procedure is carried out. The middle turbinate, positioned more deeply within the nasal cavity, assumes a concealed position that necessitates the removal of the lower turbinate to reveal it during the endoscopic examination, allowing for its comprehensive visualization and assessment.

**Figure 15:**
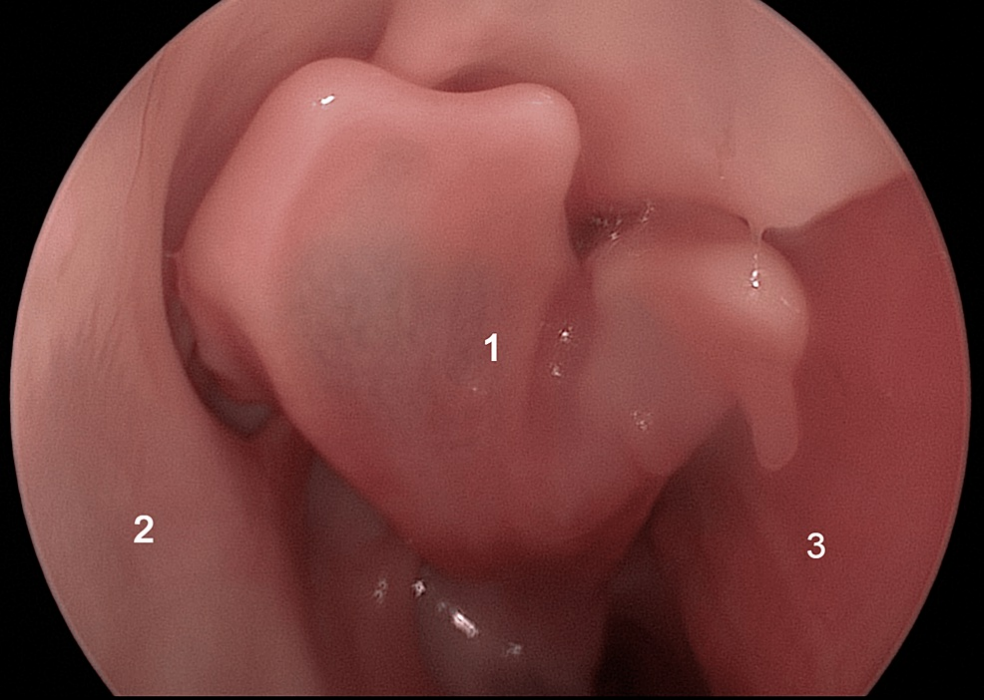
Endoscopic view: right nasal fossa 1: bifid aspect of middle turbinate; 2: lateral nasal wall; 3: septum.

By carefully excising a segment of the inferior turbinate, it becomes possible to accentuate the prominence of the middle turbinate, which serves as a crucial reference point in identifying the natural ostium of the maxillary sinus. The enhanced visibility and accentuated presence of the middle turbinate, achieved through the targeted resection of the inferior turbinate, play a vital role in identifying and locating the natural ostium of the maxillary sinus. During this stage, an additional distinctive feature comes into view: the bifid head of the middle turbinate (Figure 15).

**Figure 16:**
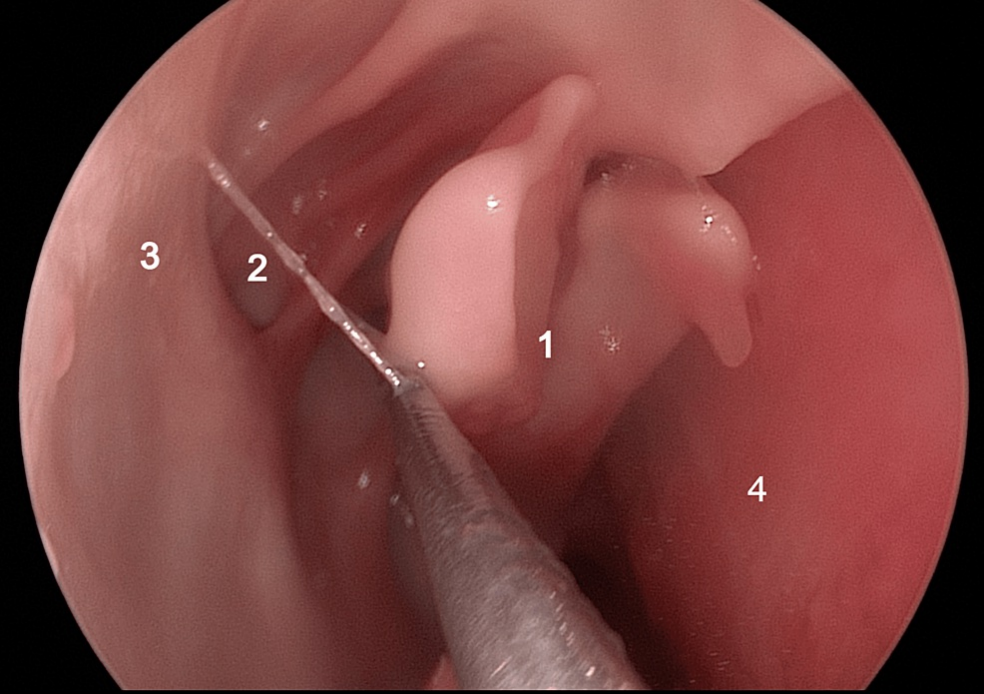
Endoscopic view: right nasal fossa 1: medialized middle turbinate; 2: natural ostium of the maxillary sinus; 3: uncinate process; 4: septum.

By gently medially displacing it using an elevator, the natural ostium of the maxillary sinus becomes visible, allowing for its comprehensive assessment (Figure 16).

**Figure 17:**
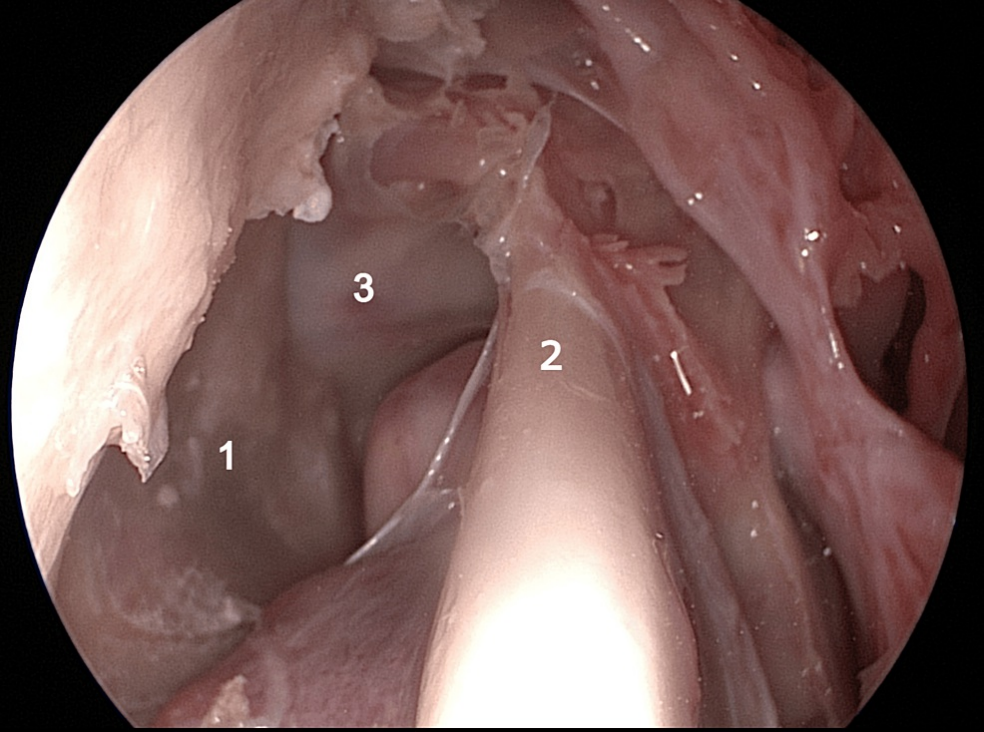
Endoscopic view: large middle antrostomy 1: maxillary sinus exposed; 2: infraorbital rim; 3: orbital wall.

After this, the uncinate process can be removed and a middle wide antrostomy can be performed with the visualization of the two sinus chambers separated by the infraorbital rim and the posterior maxillary sinus wall that corresponds to a portion of the anterior orbital wall (Figure 17).

**Figure 18:**
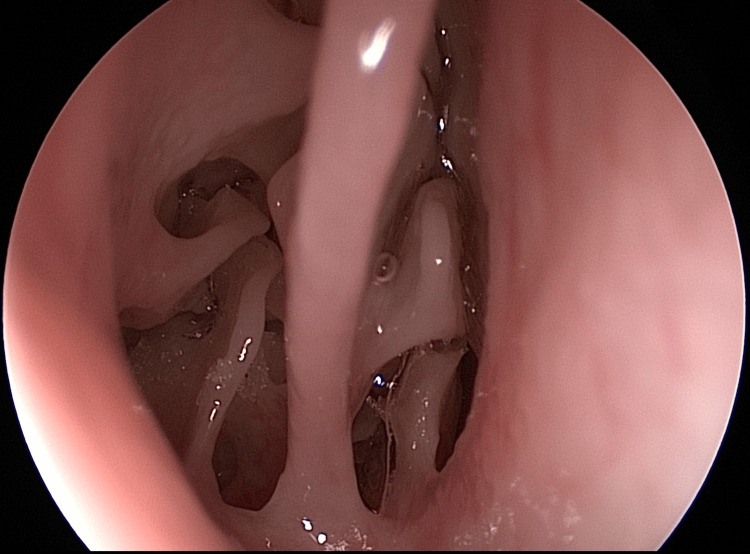
Endoscopic view: ethmoidal labyrinth

Endoscopically, the characteristic aspects of the ethmoid sinus can be confirmed, namely, it is positioning under the superior turbinate and its excessive pneumatization, aspects that can be advantageous for training procedures at the level of the ethmoid (Figure 18).

## Discussion

The use of cadaveric models for acquiring surgical skills has as a limitation the absence of real intraoperative conditions (bleeding) that can contribute to the acquisition of surgical skills. The sheep’s head model appears very suitable for endoscopic surgical maneuvers. This study showed that the sinonasal anatomy of the sheep's head presents many similarities to human sinus anatomy, making it a suitable cadaveric model for rhinologic analysis.

Finally, analysis should be made with clarity of some of the differences between sheep and human head anatomy. It should also be important that the spatial orientation and topographic particularities of important landmarks associated with complications in endoscopic sinus surgery are distinct in the sheep model when compared to human head anatomy.

Utilizing a sheep head as a surrogate for human cadaver heads in nasal surgery simulations raises intriguing possibilities within the realm of medical research. This discussion will delve into the advantages, limitations, and potential implications of such a substitution, while acknowledging the critical importance of validating the findings before widespread adoption.

One of the primary advantages of employing sheep heads lies in their anatomical similarities to human heads, particularly in the nasal region. The nasal passages, sinuses, and surrounding structures exhibit comparable features, providing researchers with a plausible model for certain aspects of nasal surgery. This similarity allows for the exploration of various surgical techniques, instruments, and interventions in a controlled environment.

The histological findings imply a significant level of comparability between the sheep and human mucosal characteristics. It is remarkable to observe parallelism in the fundamental histological features and overall mucosal architecture between sheep and human nasal tissues.

Regarding the ethmoidal roof, type 3 identified in the studied ovine model, is particularly critical in endoscopic sinus surgery due to its thin cribriform plate, making it the most challenging and significant type. According to the Keros classification, the depth of the olfactory fossa can be categorized into three types: type 1 (1-3 mm), type 2 (4-7 mm), and type 3 (8-16 mm) [14].

Furthermore, the availability and cost-effectiveness of sheep heads make them an attractive alternative to human cadavers. Obtaining human cadavers for research purposes can be logistically challenging, and ethical considerations add an additional layer of complexity. Sheep heads offer a readily accessible and ethically sound substitute that could facilitate more extensive and varied studies in nasal surgery.

However, it is crucial to acknowledge the limitations associated with using sheep heads instead of human cadaver heads. While there are anatomical similarities, there are also notable differences that must be considered. The inferior nasal turbinate, consisting of two main portions, upper and lower, is similar to what Skitarelić and Mladina identified in his study on the lamb's head [15], an aspect that can be a little confusing at first and can sometimes be confused with the middle turbinate.

Also, a characteristic of the ovine model confirmed by other authors, is the absence of the sphenoid sinus, which is explained by the lack of natural angulation of the skull base, an anatomical peculiarity commonly found in quadrupeds [15,16].

The size, shape, and orientation of certain structures may vary, potentially influencing the outcomes of surgical simulations. Researchers must exercise caution and interpret results with a clear understanding of these anatomical distinctions.

Furthermore, the translational potential of findings from sheep head studies to human clinical practice warrants scrutiny. While sheep heads may offer a valuable initial platform for testing and refining surgical techniques, the ultimate goal is to apply these insights to benefit human patients. Researchers must bridge the gap between sheep models and human applications, considering factors such as tissue response, healing mechanisms, and long-term outcomes.

## Conclusions

In conclusion, the use of sheep heads as substitutes for human cadaver heads in nasal surgery simulations presents a promising avenue for research. The anatomical similarities and cost-effectiveness make sheep heads a practical choice for certain aspects of endoscopic sinus surgery training. However, researchers must approach this methodology with a thorough understanding of its limitations, including anatomical and biomechanical differences. Validation studies comparing outcomes between sheep and human models are essential to establish the reliability and translational potential of findings. While sheep heads offer a valuable resource, the path forward involves meticulous research, validation, and a cautious approach toward applying insights to human clinical practice.

The sheep’s head anatomical model provides highly valuable experience for young trainees in endoscopic sinus surgery. Despite encountering several challenges, including some anatomical differences, considering its advantageous attributes renders it an ideal material for mimicking surgical procedures in FESS.
